# Drug-induced cell viability prediction from LINCS-L1000 through WRFEN-XGBoost algorithm

**DOI:** 10.1186/s12859-020-03949-w

**Published:** 2021-01-06

**Authors:** Jiaxing Lu, Ming Chen, Yufang Qin

**Affiliations:** College of Information Technology, Shanghai Ocean University, Hucheng Ring Road, Shanghai, China

**Keywords:** Cell viability, Drug sensitivity, Perturbation signatures, WRFEN-XGBoost algorithm

## Abstract

**Background:**

Predicting the drug response of the cancer diseases through the cellular perturbation signatures under the action of specific compounds is very important in personalized medicine. In the process of testing drug responses to the cancer, traditional experimental methods have been greatly hampered by the cost and sample size. At present, the public availability of large amounts of gene expression data makes it a challenging task to use machine learning methods to predict the drug sensitivity.

**Results:**

In this study, we introduced the WRFEN-XGBoost cell viability prediction algorithm based on LINCS-L1000 cell signatures. We integrated the LINCS-L1000, CTRP and Achilles datasets and adopted a weighted fusion algorithm based on random forest and elastic net for key gene selection. Then the FEBPSO algorithm was introduced into XGBoost learning algorithm to predict the cell viability induced by the drugs. The proposed method was compared with some new methods, and it was found that our model achieved good results with 0.83 Pearson correlation. At the same time, we completed the drug sensitivity validation on the NCI60 and CCLE datasets, which further demonstrated the effectiveness of our method.

**Conclusions:**

The results showed that our method was conducive to the elucidation of disease mechanisms and the exploration of new therapies, which greatly promoted the progress of clinical medicine.

## Background

In recent years, the study of cell death process has always been the hot topics in biology and medicine [1]. With the development of cell biology and molecular biology, the mechanism of cell death has gradually been revealed. Programmed cell death was induced by many factors, including external factors such as radiation, drugs and viral infections, and internal factors such as tumors, autoimmunity and degenerative diseases [2]. It has been reported that the cell viability mechanism could be used to stimulate and inhibit the apoptosis of tumor cells through the action of the compounds. Changes in the proportion of apoptosis and abnormal behavior of cell proliferation are highly correlated with compound concentration and perturbation time, which is one of the key factors for the formation and development of tumor cells [3]. With the emergence of more canceromics data, it is still a challenge to apply cell activity mechanisms to design the best intervention strategy for the duration of the drug action, and to construct a cell signaling model to interpret these data and make accurate predictions [4].

Cell perturbation signatures are closely related to the cell viability with the action of the compounds. In the study of drug sensitivity and anticancer drug response prediction, we can predict cell phenotypes from different high-coverage molecular data since compounds control the expression and function of target proteins or enzymes in the apoptotic pathway and induce abnormal cell apoptosis. Because clinical collection of experimental data on patient and drug interactions are expensive and impractical, it was expected that the preclinical prediction models based on large-scale pharmacogenomics of cancer cell lines could be applied. In recent years, the prediction model scheme designed by machine learning method from the perspective of cell viability research has made breakthrough progress. Based on the genomic background of each cell lines, Michael P. Menden. et al. trained a neural network model to predict its IC50 distribution throughout the cell lines [5]. Due to the high-dimensional and nonlinear nature of the omics data, Yongcui Wang et al. proposed a Bayesian Neural Network (BNN) method based on the general approximation capability of feedforward neural networks to solve this problem. Compared with the deep neural network, each model might be relatively weak, but the entire mixed model could still perform well in data fitting and prediction [6].They found that the sensitivity of cancer cells to drug molecules is driven by the characteristics of cells and drugs. Emdadi, A. and Eslahchi, C. proposed a DSPLMF method based on a recommendation system. The gene expression profile, copy number variations and single nucleotide mutation information were used to calculate the similarity of the cell lines, and the chemical structure was used to calculate the similarity of the drugs. And the possibility of cell lines being sensitive to drugs was calculated through the logical matrix decomposition to discover the effective characteristics of the cell lines and drugs [7]. Similarly, Xie et al. used a deep learning model to predict the response and efficacy of different anticancer drugs to the breast cancer, and proposed an unsupervised variational autoencoder model geneVAE and rectified junction tree variational autoencoder (JTVAE). GeneVAE and JTVAE were found to have strong robustness in drug response prediction of breast cancer cell lines and whole cancer cell lines [8]. Su, Ran et al. used genetic information, chemical characteristics and biological context with the ensemble optimization strategy, and combined with the weighted model META-GDBP to predict drug response, which found a high correlation between predicted drug response and observed drug response [9]. Sharifi-Noghabi Hossein et al. proposed a deep neural network MOLI algorithm, which took somatic mutation, copy number variation and gene expression as input data and used a combination of multi-omics methods and clinical data to predict drug response. Compared with the latest single-omics and early integrated multi-omics methods, their proposed method had a significant improvement in prediction performance [10]. Similarly, Szalai Bence et al. conducted a model prediction analysis based on the correlation between the differentially expressed genes measured in the cell lines and the drug sensitivity under the action of the the drug at a specific concentration, and found that the cell line response was correlated with the drug concentration and time. However, the model achieved low accuracy and poor fitting in the prediction process because it ignored the non-linear characteristics between differentially expressed genes and the drug sensitivity [11].

In this study, we developed the WRFEN-XGBoost algorithm to predict the cell viability under the drug induction using LINCS-L1000 perturbation signatures. Firstly, we screened and matched the three data sets, including perturbation transcriptomics signatures (LINCS-L1000), cancer treatment response portal (CTRP) and cancer dependence map database (Achilles), and divided them into nine data subsets. Secondly, we proposed a weighted fusion algorithm based on random forest and elastic nets to effectively extract non-linear features between differentially expressed genes and cell viability, and completed the selection of key genes. Then, we used the XGBoost algorithm to predict the cell viability and analyzed the apoptosis response under the action of drug toxicity and gene silencing. At the same time, in order to avoid the problem of tedious parameter adjustment, we introduced the FEBPSO algorithm into the XGBoost learning algorithm. Finally, in order to measure the feasibility of our method, we completed cross-dataset validation between compounds and shRNAs at different perturbation times. In addition, we validated the drug sensitivity inference on the two benchmark data sets of CCLE and NCI60.

## Methods

### Dataset collection

We used five datasets in this study, including the perturbation transcriptomics signatures (LINCS-L1000), the Cancer Therapeutics Response Portal (CTRP), the Cancer Dependence Map Database (Achilles), the Cancer Cell Line Encyclopedia (CCLE) and NCI-60 dataset. LINCS adopted L1000 technology to detect the transcriptome expression data in human cancer cell lines under various external stimulation. The expression of the whole genome was extrapolated by detecting the expression levels of 978 genes [12, 13]. The differentially expressed signatures corresponding to level five in the LINCS project were chosen as the training data set, and the data could be obtained from the website https://www.ncbi.nlm.nih.gov/geo/. To analyze the cellular response of the cancer cell lines to specific therapeutic drugs, we used the Cancer Treatment Response Portal (CTRP), which covered the link between compound sensitivity and genetic or lineage characteristics in 70,000 cancer cell lines. We selected post-quality-control cell viability values as a target for our modeling, which could be downloaded from the website https://ocg.cancer.gov/programs/ctd2/data-portal [14]. The third dataset, Cancer Dependence Map Database, could be obtained from the website https://portals.Broadinstitute.org/achilles and we selected the log fold scores of effects change before and after shRNA treatment for our model analysis [15].

To verify the effectiveness of our prediction model, we used the NCI-60 dataset and the Cancer Cell Line Encyclopedia (CCLE) as validation datasets in the end, respectively. The NCI-60 dataset could be downloaded from website https://dtp.cancer.gov/discovery_development/nci-60, and we set GI50 value as the evaluation standard for drug sensitivity [16]. The last dataset was the CCLE dataset, which consisted of the responses of more than 400 cell lines and 24 compounds at eight concentration points, as well as the expression data of 18,926 genes for each cell line. The CCLE dataset could be downloaded from the website https://portals.broadinstitute.org/ccle, and we used the active area of the drug as the evaluation standard for drug sensitivity [17].

### Dataset preprocessing

We first merged the two-stage perturbation screens LINCS-L1000-PhaseI and LINCS-L1000-PhaseII, and obtained the genome-wide gene expression levels under various perturbations in LINCS-L1000. To further analyze the cell viability of different cell lines under the compound perturbation, we correlated it with the cell viability data after drug treatment in CTRP. We matched the sample instances based on the same cell line and the drug identification number provided by the Broad Institute. We referred to (1) to match samples with similar concentrations. For different experimental batches, we took the average value of the cell viability which was measured in the same concentration.

1 $$\begin{aligned} doseDiff=\vert log_{10} (Cdose)-log_{10} (Ldose) \vert \le 0.2 \end{aligned}$$

where *Cdose* was the concentration value corresponding to the cancer treatment drug in CTRP, and *Ldose* was the concentration value corresponding to the perturbation signatures in LINCS-L1000.

**Fig. 1:**
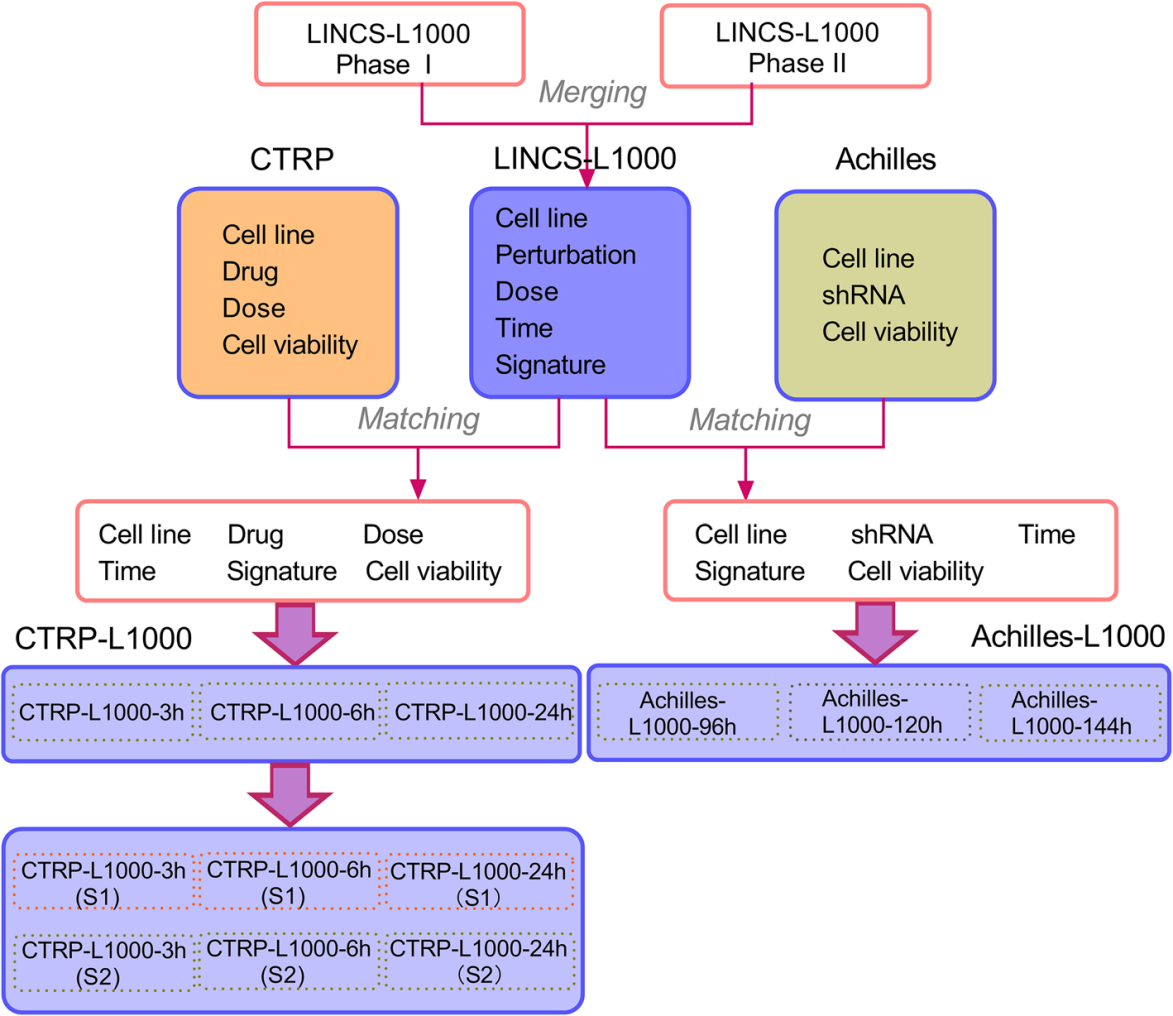
LINCS-L1000 and CTRP, Achilles data association diagram. The process of data association consisted of two parts: perturbation signatures and cell phenotypic information. The LINCS-L1000-PhaseI and LINCS-L1000-PhaseII were combined and renamed LINCS-L1000. The compound perturbation signatures and shRNA perturbation signatures involved in LINCS-L1000 were respectively associated with CTRP and Achilles datasets according to relevant conditions, which were named CTRP-L1000 and Achilles-L1000. The datasets were divided into CTRP-L1000-3h, CTRP-L1000-6h, CTRP-L1000-24h, Achilles-L1000-96h, Achilles-L1000-120h and Achilles-L1000-144h according to different perturbation time. CTRP-L1000-3h, CTRP-L1000-6h, and CTRP-L1000-24h were divided into six subsets according to the concentration factor was considered(S2) or not considered(S1)

In the course of the research, in order to enable our training model to be tested independently on other datasets to verify the effectiveness of the model, we attempted to use similar phenotypic information to the cancer treatment response portal CTRP for further research. We associated the merged two-stage LINCS-L1000 perturbation screen data with the Achilles project, the cancer dependency map database, to investigate the effect of single gene knockdown or knockout on apoptosis or proliferation of cancer cells under the action of shRNA. Since the number of cell survival after drug treatment or shRNA treatment was proportional to the evaluation indicators in the CTRP project or the Achilles project, for simplicity, we referred to the cell phenotypic information in the above two data sets as cell viability. The specific process above was shown in Fig. 1.

### Model establishment

**Fig. 2:**
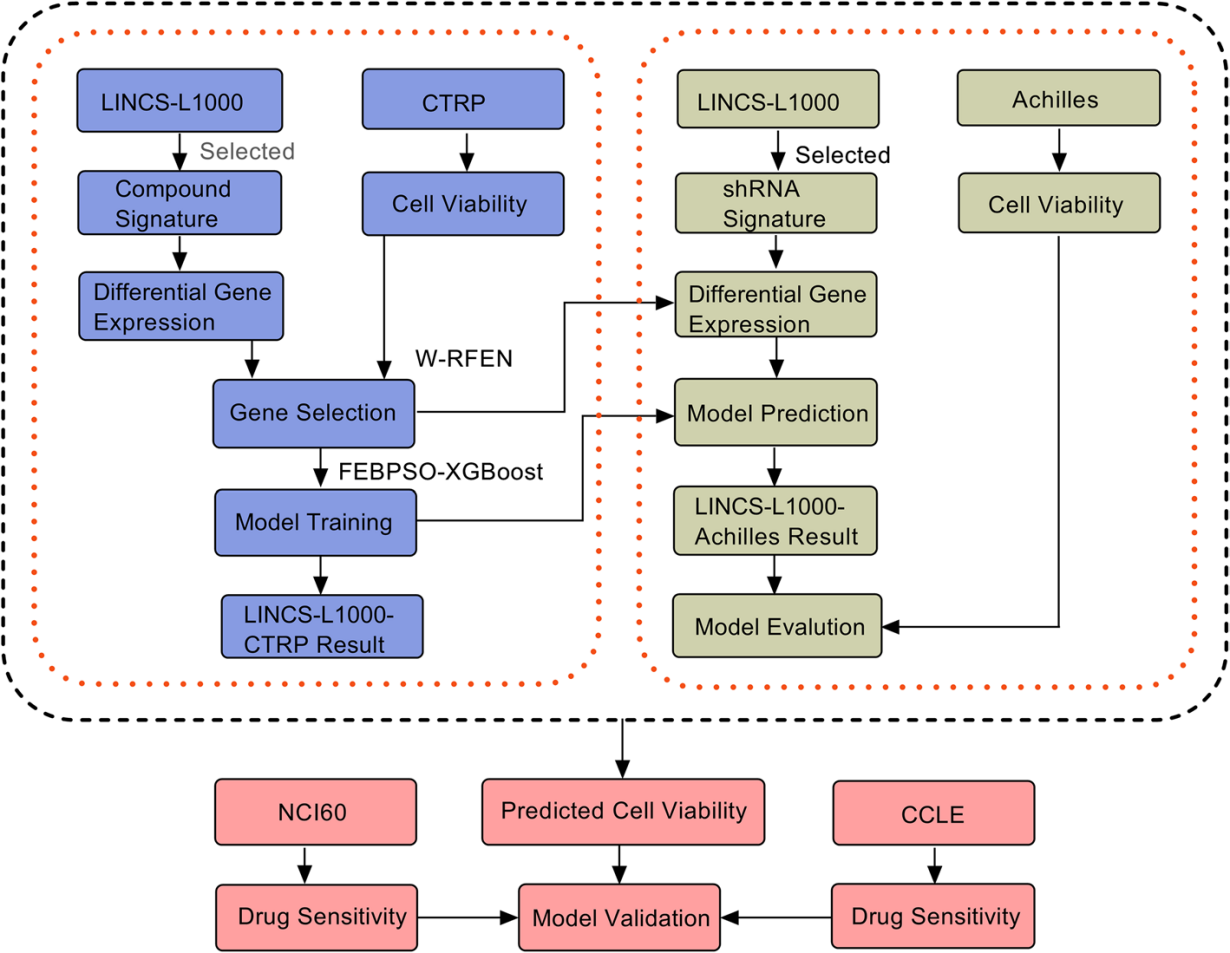
Framework diagram of cell viability prediction

The research framework of this study is shown in Fig. 2. In the first place, we completed the selection of differentially expressed genes and predictive analysis of cell viability on the perturbation transcriptomics signatures LINCS-L1000 and the cancer treatment response portal CTRP dataset. To derive the model’s performance across the datasets, we then performed independent screen tests on the cancer dependency map database Achilles (only the test process of the CTRP-L1000 model on the data set Achilles-L1000 is presented here, and vice versa). At the same time, we conducted the model validation based on the active area value in the Cancer Cell Line Encyclopedia CCLE dataset and the drug sensitivity index in the NCI-60 dataset.

#### Feature extraction based on random forest and elastic net

Random forest, as a typical representative of the Bagging method in ensemble learning, can guarantee the improvement of the regression accuracy and search for a large number of non-linear features [18]. It is considered as one of the most successful algorithms to describe the correlation between key genes and cell phenotype studies [19]. In this study, the sample space is randomly divided into different parts by bootstrapping method. For each node of the decision tree, several genes are randomly selected from the M-dimensional differentially expressed gene space $\varvec{OriDEGs}=(\varvec{g}_1,\varvec{g}_2,\varvec{g}_3, ,\varvec{g}_M)$ and then form the Z-dimensional gene subspace $\varvec{SubGenes}=(\varvec{i}_1,\varvec{i}_2, \varvec{i}_3, ,\varvec{i}_Z)$. Then we select the best split node and get the result of the sample by the weak decision tree. To obtain the final results, prediction of each weak decision tree is averaged. After obtaining the prediction results, we used the Pearson correlation coefficient to evaluate the performance of the random forest to prepare for the feature-weighted fusion. We arranged each attribute in descending order according to the importance of the genes. The non-contributing genes were removed and the number of remaining genes were recorded after sorting.

Elastic network regression, as a combination of ridge regression and lasso regression, can not only reduce the prediction variance but also achieve the purpose of coefficient shrinkage and variable selection [20]. Therefore, we use elastic net regression to select the key genes. We used the Pearson correlation on the validation set to select the appropriate parameter settings for the model. We evaluated the contribution of each characteristic gene in the model and ranked them in descending order of gene contribution.

In order to screen out effective differentially expressed genes (DEGs), we used a weighted fusion algorithm of the random forest and elastic network (referred as WRFEN) to select key genes.

2 $$\begin{aligned} W(DEGs)_{Rank}=\frac{e^{RFPearson}*(DEGs)_{Rank}^{RF} +e^{ENPearson}*(DEGs)_{Rank}^{EN}}{e^{RFPearson}+e^{ENPearson}} \end{aligned}$$

where *RFPearson* and *ENPearson* are the Pearson correlation on the validation set using random forest and elastic network algorithms. $(DEGs)_{Rank}^{RF}$ and $(DEGs)_{Rank}^{EN}$ are the feature importance order of the differentially expressed gene DEGs and the number of genes selected in the random forest and elastic network algorithm, respectively.

We ranked the key genes in the random forest and elastic network respectively, and use (2) to perform weighted summation. Finally, we ranked the result and the optimal number of genes in order of gene contribution. The algorithm flowchart was shown in Additional file 1: Fig. S1. More precisely, it was a feature selection method based on the combination of random forest and elastic net. It calculated the order of each gene in two methods and the performance of the two methods in the prediction performance (Pearson correlation) was used as the weight. If the prediction performance of the model was better, the more weight it occupied in gene ranking and the higher the genes in the final ranking.

#### Cell viability prediction algorithm based on XGBoost and FEBPSO

XGBoost is one of the most competitive prediction algorithm in machine learning. It improves the integration of the gradient boosting algorithm and has high performance in solving both classification and regression problems [21]. We used the XGBoost algorithm to predict cell viability and obtained a prediction score on the leaf node of each decision tree based on the differential expression of genes in each sample. Multiple weak estimators are constructed one by one through multiple iterations. The cell viability prediction result is defined as the sum of the prediction scores of all the trees as follows.

3 $$\begin{aligned} \hat{cv_i}=\sum _{k=1}^Kf_k(sample_i[DEGs]) \end{aligned}$$

where $f_k(sample_i[DEGs])$ represents the prediction score on the *k*-th decision tree for the *i*-th sample on the selected differentially expressed gene set DEGs. *K* is the number of decision trees. Then during the *t*-th iteration of the sample, the model’s predicted value $\hat{cv_i}$ can be described as follows:

4 $$\begin{aligned} \hat{cv_i}^{(t)}=\hat{cv_i}^{(t-1)}+f_t(sample_i[DEGs]) \end{aligned}$$

In this study, in order to improve the prediction accuracy of cell viability and reduce the prediction bias, we used the discrete binary particle swarm optimization with flexible weights algorithm FEBPSO to adaptively adjust the parameters of XGBoost. As a typical representative of swarm intelligence algorithms, particle swarm optimization can effectively solve nonlinear continuous optimization problems [22]. Meanwhile, it solves the problem of too long training time due to a large amount of adjustment parameters [23]. In the prediction process of FEBPSO-XGBoost, we first initialized the binary particle swarm, encoded each parameter as a binary number and transformed the parameter optimization into a discrete combinatorial optimization problem. During each iteration, the parameters were converted into decimal numbers within the specified range in a group of six. At this time, we calculated the Pearson correlation coefficient of each individual particle running in XGBoost algorithm and evaluated the fitness of each individual particle. For each particle, we compared the current fitness value with the individual’s historical best position or global best position. If the current fitness value was higher, the historical best position and global best position would be updated with the current position of the particle. At the same time, the particle speed and position information would be updated to enter the next iteration until the termination condition has been met. Finally, the global optimal value and the best parameter settings would be output at this time. The particle speed is updated as follows:

5 $$\begin{aligned} v_i^{k+1}=wv_i^k+c_1r_1(x_{pbest,i}^k-x_i^k)+c_2r_2(x_{gbest}^k-x_i^k) \end{aligned}$$

where $v_i^k$ represents the velocity vector of particle *i* during the *k*-th iteration, $x_i^k$ represents the position vector of particle *i* during the *k*-th iteration, $c_1$ and $c_2$ are the acceleration constant, $r_1$, $r_2$ are the random number, *w* is the inertial weight, $x_{pbest,i}^k$ denotes the best position of the individual particle and $x_{gbest}^k$ denotes the best position of the global particle.

In order to overcome the shortcomings of premature convergence and falling into local extremes of particle swarm optimization, we used the formula shown below to update the weights [24].

6 $$\begin{aligned} w(k)=\alpha _1e^{\frac{-\psi *k}{T}}+\alpha _2e^{\frac{\psi *k}{T}} \end{aligned}$$

where $\alpha _1=\frac{w_2e^{\psi }-w_1e^{2\psi }}{1-e^{2\psi }}$, $\alpha _2=\frac{w_1-w_2e^{\psi }}{1-e^{2\psi }}$, *T* denotes the maximum number of iterations, *k* is the current number of iterations, $w_1$,$w_2$ are the minimum inertia weight and maximum inertia weight greater than zero, respectively.

We used WRFEN for core gene selection and FEBPSO-XGBoost for predictive analysis. Through this, we formed a complete prediction model and explained the complete apoptotic levels observed in cell lines with specific drugs and concentrations.

## Results

Based on the latest transcriptomic perturbation screens in LINCS-L1000, we conducted the study with the cell viability after the drug treatment in CTRP and the effect change score before and after the treatment with shRNA in the Achilles project, respectively. From the perspective of gene regulation, we examined the relationship between key genes and drug response. At the same time, the FEBPSO-XGBoost machine learning algorithm was used to predict the cell viability of different cell lines with the treatment of various drugs or shRNA by using the expression levels of characteristic genes under the action of different perturbation times and different drug concentrations.

### Analysis of feature selection

In the feature selection process, we firstly selected 40 trees for the establishment of a random forest, and the results were ranked according to the variable contribution. Secondly, the ratio of the lasso penalty term was set to 0.1, 0.2,0.5,0.7,0.95,1 and the coefficient penalty term was controlled to from 0.1 to 1.0 by step 0.1 in the elastic net. The best combination of the parameters was decided on the validation set. Then, we sorted the variables according to their contribution and deleted the non-contributing genes. Finally, we calculated the selected characteristic genes according to Formula (2), and obtained the final genes. The feature genes selected on each subset (subset names were shown in Additional file 1: Table S1) was ranked according to their contribution. We listed the number of feature genes selected and the contribution ranking of the fifteen key genes in each subset in Additional file 1: Table S1.

Taking the LINCS-L1000-CTRP-24h dataset as an example, we compared the WRFEN with the existing traditional methods FTest [25], MI [26], RFFS [27] and LRFS [28], and tested it on multiple predictors at the same time (Additional file 1: Fig. S2). The results showed that the results of the gene selection algorithm in this paper were better than the existing single algorithms. It could also be observed that the prediction performance of the model would be gradually stabilize as the number of selected feature genes increases.

**Fig. 3:**
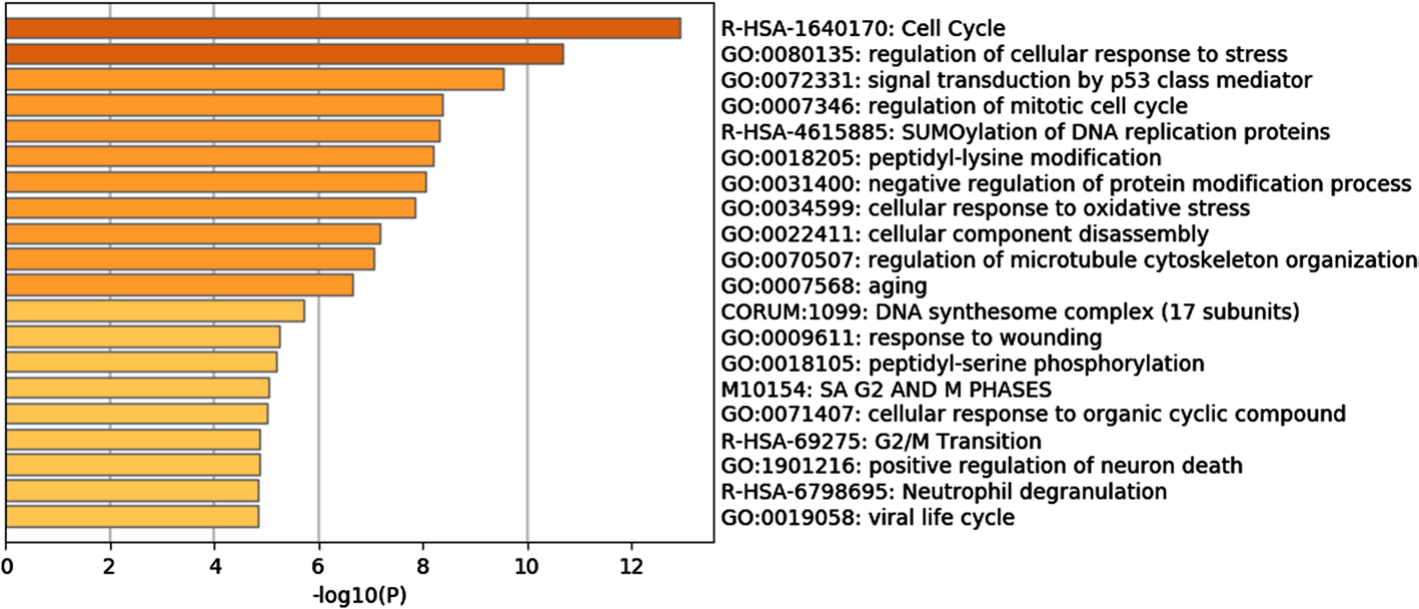
Enrichment analysis of differentially expressed genes in the CTRP-L1000-24h dataset

In order to further understand the biological functions performed by the selected characteristic genes, we took the subsets of CTRP-L1000-24h and Achilles-L1000-96h as examples to perform analysis on the extracted characteristic genes. We could find that they were all closely related to the apoptotic process from Fig. 3 and Additional file 1: Fig. S3. The most significantly enriched pathways, r-has-1640170 and GO:0007346, were involved in the regulation of cell cycle and apoptosis, which also confirmed that the differentially expressed genes selected in this study after treatment with drugs or shRNA constituted the pathway of apoptosis.

### Prediction and analysis of drug induced cell viability

**Fig. 4:**
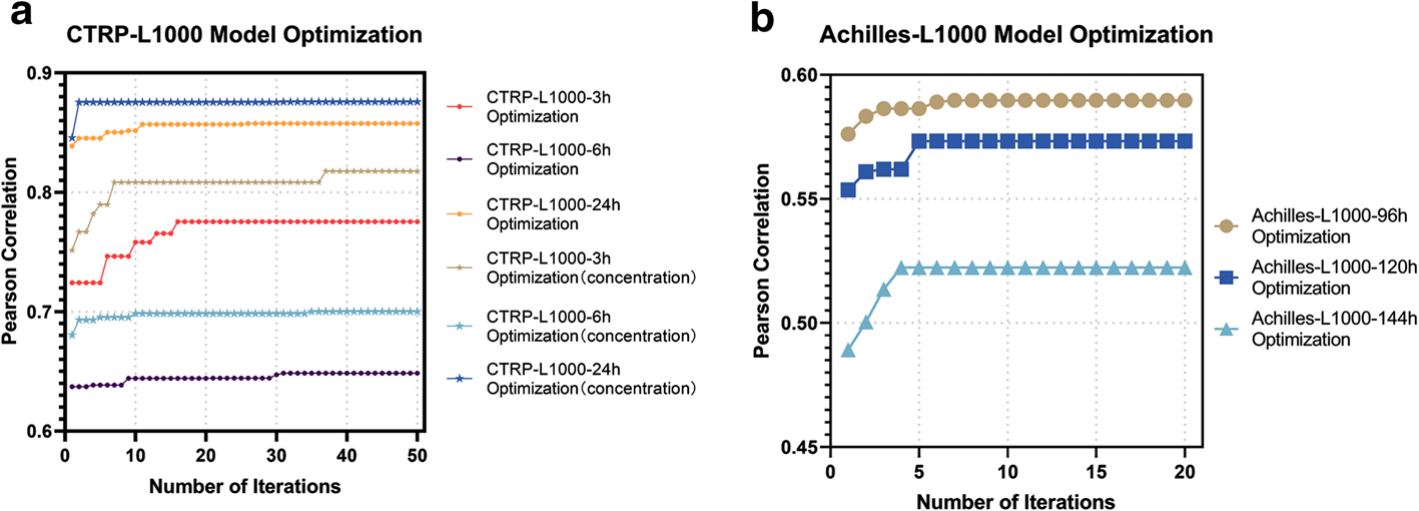
Iterative process of FEBPSO in XGBoost algorithm. **a** CTRP-L1000 Optimization. **b** Achilles-L1000 Optimization

We updated and adjusted the parameter combination of XGBoost with the binary discrete particle swarm optimization with flexible weight. We set the number of swarm particles to be 25, the dimension of the particles to be 48, the maximum number of iterations in CTRP-L1000 and Achilles-L1000 series models to be 50 and 20 respectively, the acceleration constants to be 1.5, the maximum and minimum values of inertia weight to be 0.8 and 0.4 respectively, the maximum and minimum values of velocity to be 10 and -10 respectively and weight updating formula of parameter $\psi$ to be 2.6. The correlation coefficient between the observed value and the predicted value was used as the model evaluation index and the fitness function. At the beginning of the particle swarm optimization algorithm, the population was generated randomly. When the iteration reached a certain number, the optimal solution or approximate optimal solution would be found with a high probability. The experimental results of parameter optimization in XGBoost by using FEBPSO algorithm were shown in Fig. 4.

From the above experimental results, it was obvious that the measurement of cell viability in CTRP required a long perturbation time. With the increasement of the perturbation time, the reliability of the forecast also continued to rise, and the prediction results of the 24-h perturbation time was more reliable. When the concentration factor was added in the prediction of the CTRP dataset, the prediction accuracy of the model could be improved, which indicated that the cell viability depended on the concentration of the drug to some extent. In the LINCS-L1000 perturbation screens and cancer dependency map database Achilles, the model produced by the 96-h perturbation time had the most significant prediction effect. It could be seen from the results that the disturbance time was not necessarily as long as possible.

In the optimization process of the CTRP-L1000 series model, when the number of iterations reached about 20 rounds, the prediction performance of the model gradually tended to be stable. In the process of Achilles-L1000 series model optimization, when the number of iterations reached about 8 rounds, the prediction performance of the model also gradually tended to be stable. After we used FEBPSO to adjust the parameters of the XGBoost model, the optimal parameter combinations and default values of each parameter were shown in Table 1 and Additional file 1: Table S2 below. It could be seen that this experiment fully proves the effectiveness of the parameter optimization algorithm proposed by this research. Compared with the traditional default parameters, using the FEBPSO algorithm to optimize the parameters of the XGBoost model had significantly improved the accuracy of model prediction.

**Table 1:** XGBoost parameters and best parameter combinations (CTRP-L1000 Series Model)

Parameter Name	L1000-CTRP-3h (S1/S2)	L1000-CTRP-6h (S1/S2)	L1000-CTRP-24h (S1/S2)
Learning rate	0.0225/0.0476	0.01/0.0225	0.01/0.035
Gamma	0/0.0317	0.1587/0	0/0
Max depth	6/3	5/5	6/5
Min child weight	4/5	3/13	8/10
Subsample	0.5757/0.7957	0.4343/0.5129	0.2457/0.6700
Colsample_bytree	0.1111/0.0794	0.4286/0.1270	0.4762/0.8095
Lambda	0.01/1.1156	1.2103/0.3259	1.4946/0.7997
Iteration times	4174/1476	5841/3460	4968/4492

### Independent dataset validation on CTRP-L1000 and Achilles-L1000

**Fig. 5:**
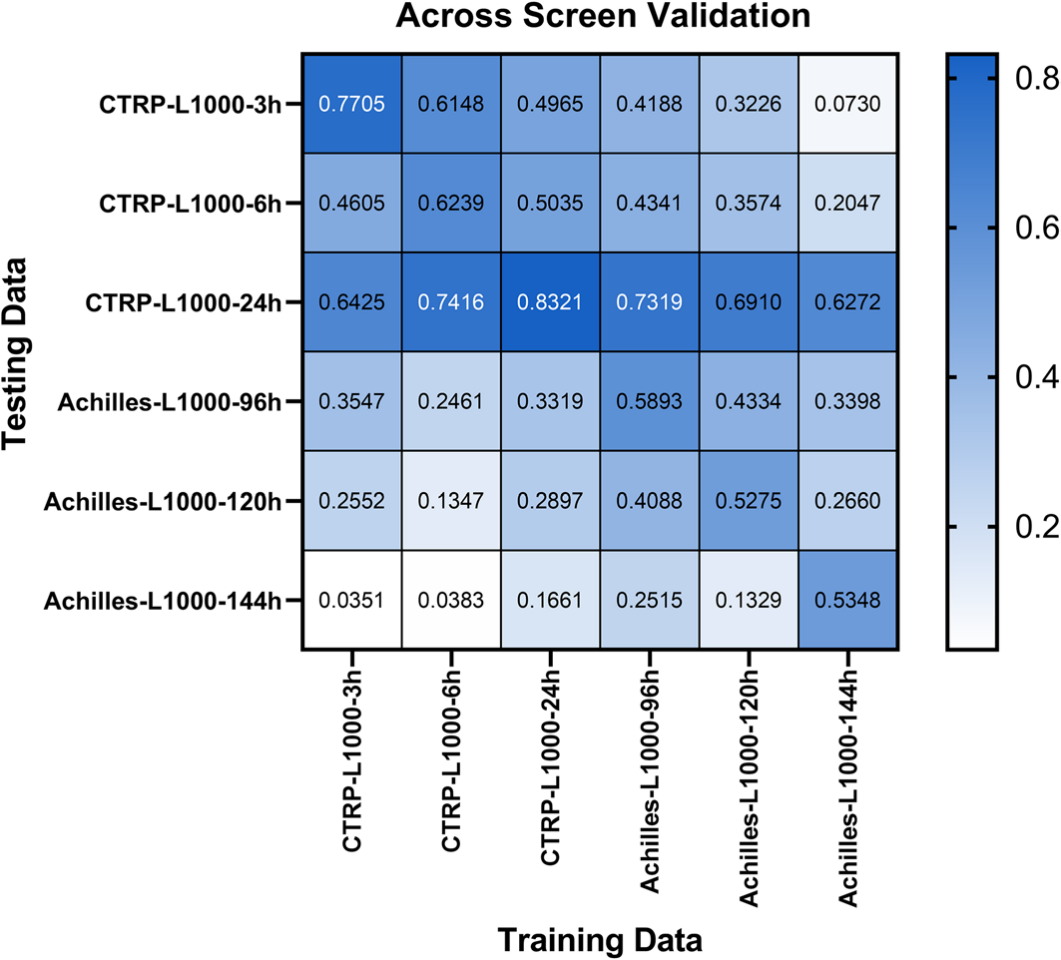
Independent dataset validation. Using the Achilles-L1000 series model to predict cell viability in CTRP-L1000 data and vice versa

In order to verify the reliability of the model predictions, we used independent datasets to verify the model’s prediction capabilities. We had implemented the interactive test in the CTRP-L1000 series model and the Achilles-L1000 series model. The Fig. 5 showed the experimental results. From the figure above, it could be found that the 24-h perturbation time was the best in the CTRP-L1000 data set. The Pearson correlation of the model on this data set was 0.8321, which was better than the 3-h and 6-h perturbation times. In the Achilles-L1000 dataset, the 96-h perturbation time was considered to be the best. The performance of the model on this data set is better than the perturbation time of 120 h and 144 h with 0.5893 Pearson correlation. Similarly, in terms of independent set validation, the CTRP-L1000-6h model, CTRP-L1000-24h model and Achilles-L1000-96h model was superior to other models in CTRP-L1000-24h screen with 0.7416, 0.8321 and 0.7319 Pearson correlation, respectively. Therefore, we further confirmed that the drug could achieve excellent predictive performance after a longer perturbation time.

### Model validation on the NCI60 dataset

In order to validate the model across the NCI60 dataset, we used the GI50 value as the indicator of drug sensitivity evaluation and binarized the GI50 value (50% growth inhibition). In the NCI60 dataset, when the efficacy was within the range of 50% growth inhibition concentration, it corresponded to the GI50 value in the drug sensitivity evaluation index. When the efficacy was not effective within the 50% growth inhibition concentration range, it was recorded as the highest concentration value. In this study, we would define the drug concentration difference variable, which portrayed the efficacy of the drugs and was calculated as shown in Formula (7). In other words, when the value of the drug concentration difference was less than zero, it meant that the drug was an effective drug, otherwise it was an ineffective drug.

7 $$\begin{aligned} \Delta drug\_conc(dr,cl)=drug\_sens(dr,cl)-test\_max\_conc(dr,cl) \end{aligned}$$

where, $\Delta drug\_conc(dr,cl)$ was the difference in drug concentration when the cell lines *cl* under the treatment of the specific drug *dr*. $drug\_sens(dr,cl)$ was the drug sensitivity value GI50 for *cl* treated by *dr*. $test\_max\_conc(dr,cl)$ was the maximum tested drug concentration used in the treatment of cell line *cl* with the drug *dr*.

**Fig. 6:**
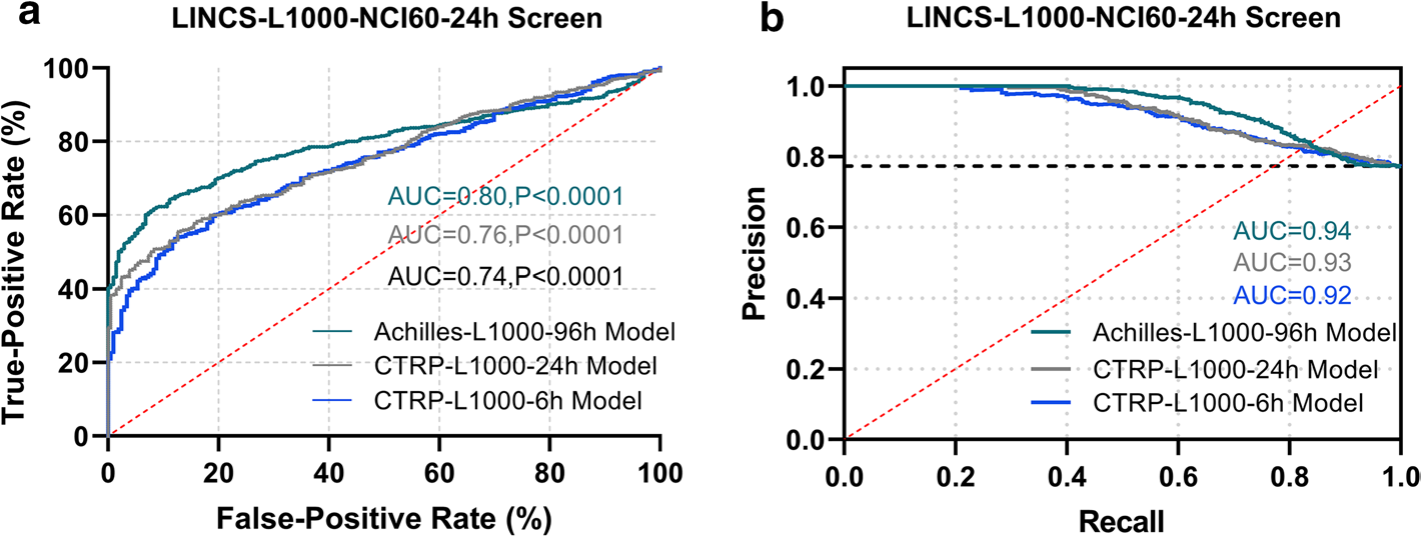
ROC curve and PR curve of the model evaluation on LINCS-L1000-NCI60-24h dataset. **a** The graph of Receiver Operating Characteristic. **b** The graph of Precision-Recall

In this study, ROC curve and PR curve were used to measure the contribution of the algorithm in evaluating the drug effectiveness. By observing the ROC curve shown in Fig. 6a, we could find that the prediction made by the Achilles-L1000-96h model is the most accurate in the LINCS-L1000-NCI60-24h dataset. When using this model for prediction, the AUC area under the ROC curve reached 0.80, the 95% confidence interval ranged from 0.769 to 0.822, and the significance level was less than 0.0001. The other two models also had good performance. Among them, the AUC area under the ROC curve of the CTRP-L1000-24h model reached 0.76, and the area under the ROC curve of the CTRP-L1000-6h model reached 0.74. In the accuracy-recall evaluation curve shown in Fig. 6b, the Achilles-L1000-96h model still surpassed other models with the area under the curve $\hbox {AUC} = 0.94$. Through the above analysis, we further confirmed that the Achilles-L1000-96h model was effective during the prediction process of the LINCS-L1000-NCI60-24h data set, and it could be further used for the effectiveness testing of other drugs.

Furthermore, we also matched and correlated the LINCS-L1000 perturbation screens, CTRP data and NCI60 data according to the matching method described above. The drug with the perturbation time of 24 h was recorded as LINCS-L1000-CTRP-NCI60-24h. In this experiment, we used CTRP-L1000-6h, CTRP-L1000-24h and Achilles-L1000-96h models to predict the cell viability in three major data sets, which had drugs and cell lines in common. We also binarized the drug sensitivity data in NCI60.

Finally, we used the ROC curve and PR curve to discuss and analyze the experimental results. As shown in Additional file 1: Fig. S4, when we used the Achilles-L1000-96h model, the CTRP-L1000-24h model and the CTRP-L1000-6h model to predict the effectiveness of the drug, the area under the ROC curve achieved 0.78, 0.80 and 0.72, respectively, and the area under the PR curve achieved 0.98, 0.98 and 0.97, respectively. The above results indicated the superior prediction performance of the Achilles-L1000-96h model and the CTRP-L1000-24h model.

**Fig. 7:**
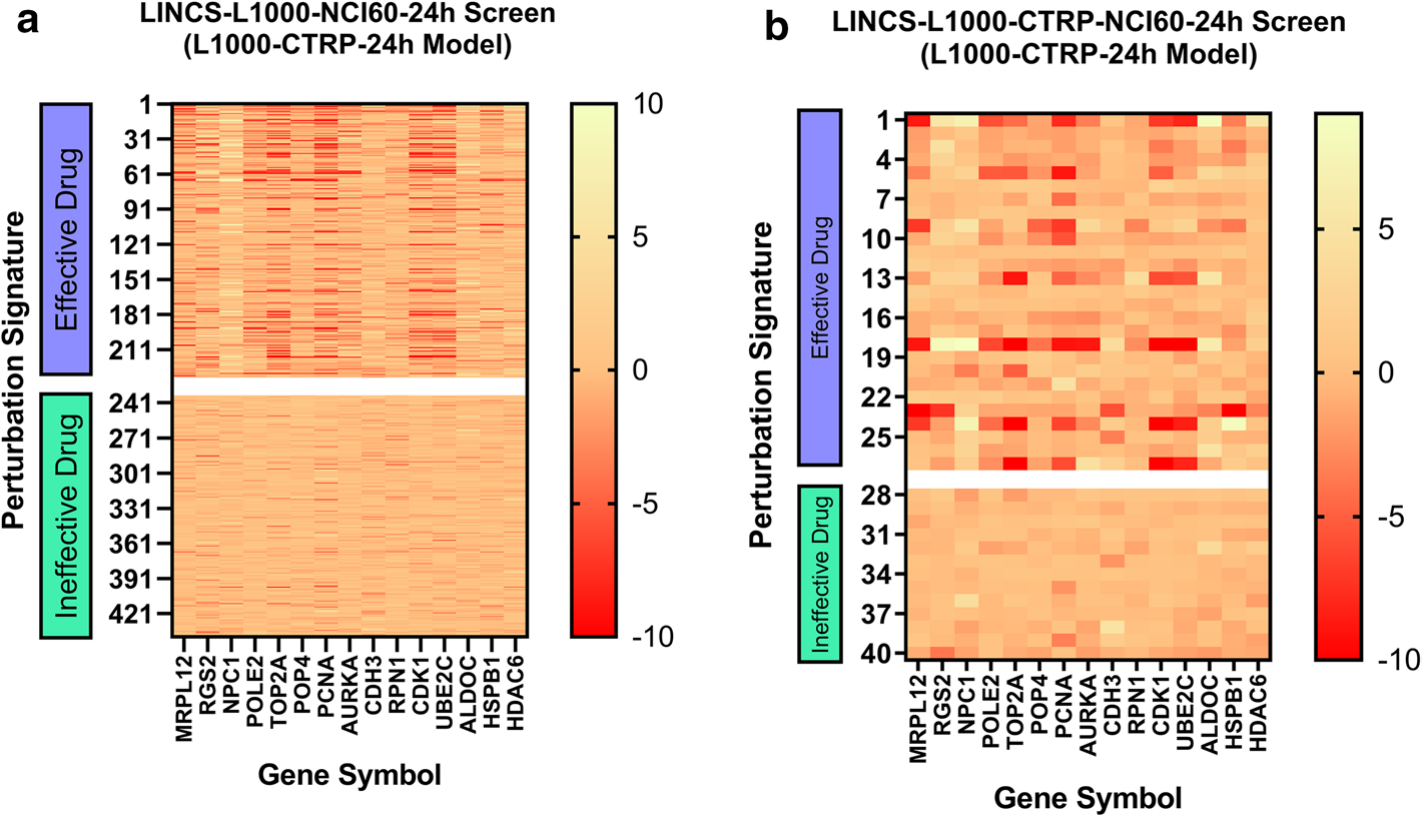
Heat map of the first fifteen genes. **a** LINCS-L1000-NCI60-24h. **b** LINCS-L1000-CTRP-NCI60-24h

While predicting the effectiveness of the drugs, we required that the predictors used in this study could make effective predictions. In addition, whether the appropriate features could be selected during the feature selection stage directly affected the predictive performance of the predictors. To do this, we correlated the selected feature genes with the effectiveness of the drug. We observed whether the differential expression levels of selected characteristic genes have significantly different expression patterns under the action of effective or ineffective drugs. For this reason, we mapped the differential expression levels of the first 15 differentially expressed genes selected in the feature selection stage under the treatment of effective drugs and ineffective drugs. Figure 7a was the result of the LINCS-L1000-NCI60-24h dataset and Fig. 7b was the result of the LINCS-L1000-CTRP-NCI60-24h dataset. By comparison, in the effective drug group, we could find that the expression level of differentially expressed genes had significantly up-regulated or down-regulated. However, in the ineffective drug group, there was no significant change in the expression level of differentially expressed genes. Therefore, we further demonstrated the validity of selected feature genes.

**Fig. 8:**
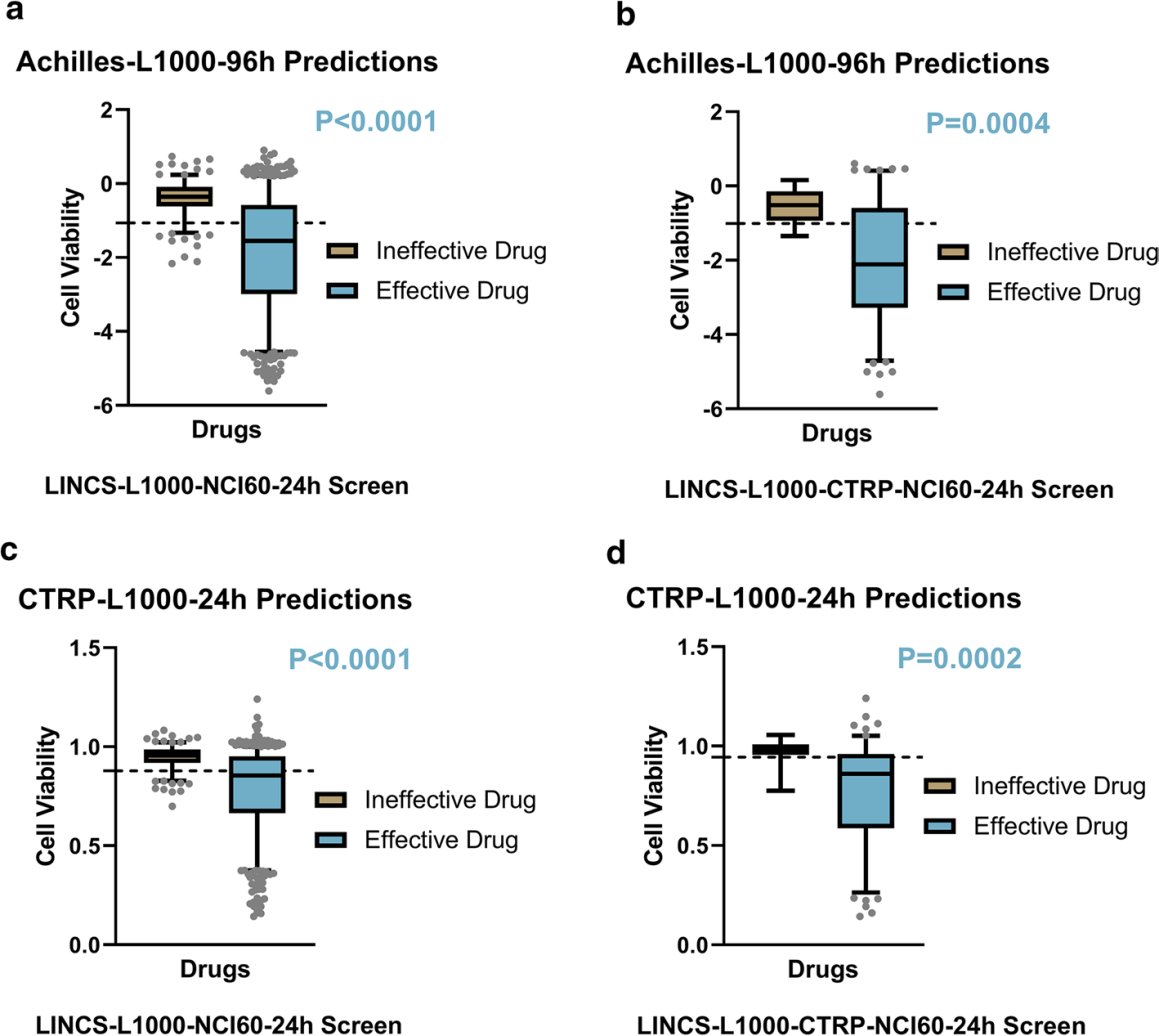
Box plot. comparison of the effective drug group and the ineffective drug group

So far, we had completed inferring the effectiveness of the drug from the predicted cell viability of each model. To further examine whether there was a significant difference between the effective and ineffective drugs on the cell viability, we used a non-parametric Mann Whitney test to analyze the cell viability prediction results, as shown in Fig. 8. Different models were predicted on LINCS-L1000-NCI60-24h screen and LINCS-L1000-CTRP-NCI60-24h screen respectively. The results found that using the Achilles-L1000-96h model to discriminate between effective drugs and ineffective drugs had a significant difference in the mean value, the significance levels were $P\le 0.0001$ and $P=0.0004$, respectively. In addition, similar results were obtained in the use of CTRP-L1000-24h model for inferring drug effectiveness, the significance levels were $P\le 0.0001$ and $P=0.0002$, respectively.

### Model validation on the CCLE dataset

Our model was also verified on CCLE, and we used the active area as the evaluation criterion of drug sensitivity. In order to achieve binarization of drug sensitivity on the CCLE data set, we first normalized the active area in CCLE to zero mean. Meanwhile, we defined the active area with 0.8 variance above the mean as an effective drug, and the active area with 0.8 variance below the mean as an ineffective drug. We then searched for common combination pairs of cell lines and drugs in the LINCS-L1000 perturbation screen. Since there were only a small number of 24 drugs in the CCLE data set, we used the PubChem database to find synonymous drugs. We marked the data after matching as LINCS-L1000-CCLE. Similarly, we screened the drugs corresponding to the perturbation time of 24 h, which were included in LINCS-L1000-CCLE-24h. At the same time, we selected the drugs whose concentration was greater than or equal to 10 micromoles. In addition, when multiple drug perturbation signatures were presented, we choose the lowest cell viability value.

We used the ROC curve shown in Additional file 1: Fig. S5(a) and the PR curve shown in Additional file 1: Fig. S5(b) to measure the results of the algorithm. By observing the experimental results, we found that when we used the drug sensitivity data in CCLE to evaluate the predicted cell viability values, the Achilles-L1000-96h model also showed excellent performance in cross-dataset validation. When we used Achilles-L1000-96h model to predict the effectiveness of the drug, the area under the ROC curve achieved 0.84 and the area under the PR curve achieved 0.88. The differential expression on effective and ineffective drugs was shown in Additional file 1: Fig. S6. We could see that the LINCS-L1000-CCLE-24h dataset still showed the same gene expression pattern as the LINCS-L1000-NCI60-24h dataset. That was to say, the differentially expressed genes in the effective drug group were significantly up-regulated and down-regulated.

## Discussion

In order to evaluate the effectiveness of the algorithm in this paper, we analyzed and compared our algorithm with other existing methods including PCA-Lasso, PCA-SVR, FTest-RF, MI-KNN, VAE [8] and DAE-NN [29]. The Principal Components Analysis (PCA), Ftest and Mutual Information (MI) were used to extract the features, and the Lasso, Support Vector Regression (SVR), Random Forest (RF) and k-nearest neighbor (KNN) were used for the final prediction. VAE and DAE-NN are proposed by the recent literature in drug response prediction. VAE used the variational autoencoder to predict the response of different anti-cancer drugs. DAE-NN used a deep autoencoder to extract the features and the neural network was for the final prediction.

In the present paper, we used the Pearson correlation coefficient, coefficient of determination ($R^2$) and mean squared error of the predicted and actual values to measure the prediction performance of the model. In the training process of VAE and DAE-NN algorithms, we used grid search to select the best training parameters for the learning rate [0.001, 0.005, 0.01, 0.05, 0.1] and iteration period [30, 90, 150, 220, 300]. The detailed experimental results of these seven algorithms were shown in Additional file 1: Tables S3–S5. Taking the CTRP-L1000-24h(S1) dataset as an example, the predicted results were shown in Table 2. Our algorithm outperformed other algorithms with the maximum correlation coefficient 0.8321, the maximum coefficient of determination 0.6922 and the minimum mean squared error 0.025.

**Table 2:** Comparison of the algorithm in this paper with other algorithms (Taking the CTRP-L1000-24h(S1) dataset as an example)

Methods	Pearson Correlation	$R^2$	Mean Squared Error
Our model	0.8321	0.6922	0.025
PCA-Lasso	0.7887	0.6211	0.031
PCA-SVR	0.7900	0.6211	0.031
FTest-RF	0.7942	0.6304	0.030
MI-KNN	0.8054	0.6414	0.030
VAE	0.8289	0.6823	0.026
DAE-NN	0.8033	0.6174	0.032

Compared with PCA-Lasso, PCA-SVR, FTest-RF, MI-KNN,VAE and DAE-NN algorithms, Pearson correlation coefficient of our method increased by 5.50%, 5.33%, 4.77%, 3.32%, 0.39%, 3.59% and $R^2$ increased by 11.45%, 11.45%, 9.80%, 7.92%, 1.45% and 12.12%. In terms of the mean squared error, our method decreased from 3.85% to 21.88% comparing with the other six algorithms above. The experimental results showed that the prediction performance of the proposed algorithm have been further improved. For the CTRP-1000-3h, CTRP-L1000-6h, CTRP-L1000-24h, Achilles-L1000-96h, Achilles-L1000-120h and Achilles-L1000-144h datasets , the evaluation results of other models were shown in Additional file 1: Tables S3–S5.

**Fig. 9:**
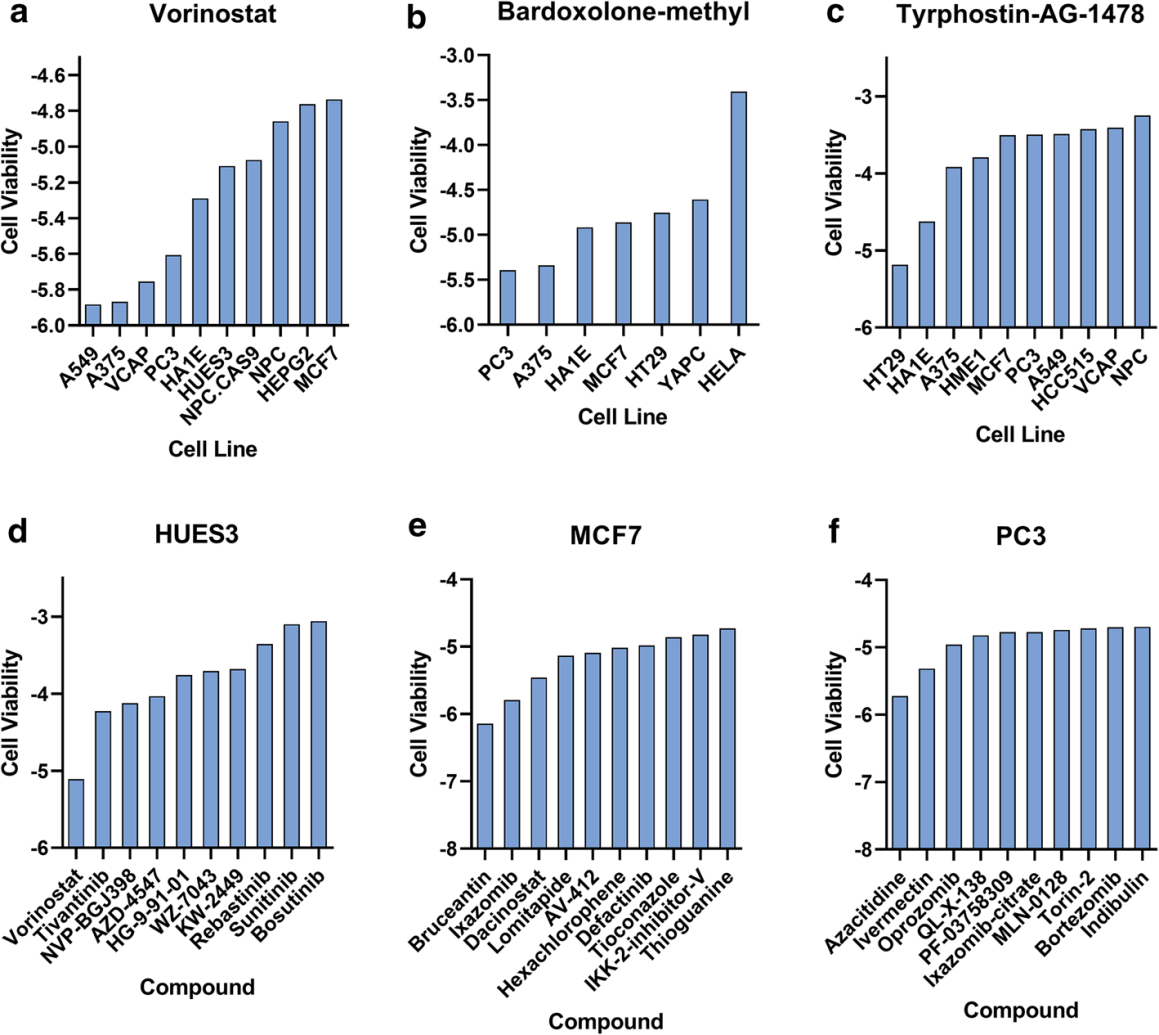
The predicted cell viability for different drugs and cell lines. **a**–**c** showed the cell viability of the drugs Vorinostat, Bardoxolone-methyl and Tyrphostin-AG-1478 in different cell lines. **d**–**f** showed the cell viability of the cell lines HUES3, MCF7, PC3 in different drugs

In addition to reliably and effectively inferring cell viability through the predictive models, we also needed to correlate our results with the literature on cell viability, as shown in Fig. 9. As a member of tumor necrosis factor receptor superfamily, high affinity nerve growth factor receptor p75NTR could induce apoptosis and inhibit the growth of prostate epithelial cells. Azacitidine-mediated p75NTR had anti-tumor effects on androgen-independent prostate cancer cells 22Rv1 and PC3 [30]. After Bortezomib treatment, the cells with suppressed C/EBPbeta levels showed delayed autophagy activation. The growth of the PC3 cells and xenografts has been decreased with the C/EBbeta gene knockdown, which could make PC3 cells sensitive to Bortezomib [31]. Another study has tested the effects on three related human glioma cell lines treated by the new epidermal growth factor receptor (EGFR) tyrosine kinase Tyrphostin-AG-1478, and found that AG-1478 was the relatively specific inhibitor of truncated EGFR. They had important medical significance because the truncated EGFR occurred frequently in glioblastoma, breast, lung and ovarian cancer [32].

## Conclusions

In this paper, we managed to predict the drug-induced cell viability from the differential gene expression data through the WRFEN-XGBoost algorithm. The study of cell phenotype was firstly correlated with the drugs and shRNA perturbation signatures. In addition, we have completed the selection of key genes based on the WRFEN algorithm and proposed a novel FEBPSO-XGBoost machine learning method to predict the cell viability. Through the connection between cell viability and pharmacogenomics, the establishment of the prediction model trained from perturbation transcriptomics signatures, cell phenotype and drug response data has been completed. At the same time, the robustness and effectiveness of our proposed modeling strategy in drug sensitivity analysis were verified on CCLE and NCI-60 datasets. This study could provide help for the biomedical researchers in drug screening and promote the analysis of anticancer drugs in pharmacogenomics.

However, in the clinical application of cancer cell lines and anticancer therapies, it is urgent to identify the biomarkers that can distinguish between drug-sensitive cell lines and drug-resistant cell lines. Firstly, besides gene expression, drug characteristics can be integrated into the model to achieve better accuracy. Secondly, a more appropriate supervised machine learning algorithm is hoped to be designed to reveal the sensitivity between cancer cell lines and drug treatment. Finally, we will continue to reveal new biomarkers that are sensitive and resistant to the cancertherapies. It provides more opportunities for exploring the biological behavior of cancer cell lines at the cellular level, and it is also the direction of our future research.

## Supplementary Information

**Additional file 1**. Supplementary Material contains supplementary figures and supplementary tables of the results in this study.

## Data Availability

The screening data (LINCS-L1000,CTRP,Achilles,NCI60 and CCLE) used to train the machine learning model presented in this study are available at https://www.ncbi.nlm.nih.gov/geo/, https://ocg.cancer.gov/programs/ctd2/data-portal, https://portals.Broadinstitute.org/achilles, https://dtp.cancer.gov/discovery_development/nci-60 and https://portals.broadinstitute.org/ccle, respectively. Source code is available at https://github.com/RuyiMz/SJZY.git. The results of the data analysis could be obtained from the additional files.

## Abbreviations

LINCS: Library of integrated network-based cellular signatures
GEO: Gene Expression Omnibus
CTRP: Cancer treatment response portal
NCI: National cancer institute
CCLE: Cancer Cell Line Encyclopedia
WRFEN: Weighted fusion algorithm of the random forest and elastic network
FEBPSO: Binary particle swarm optimization with flexible inertia weight
XGBoost: Extreme Gradient Boosting
PCA: Principal Components Analysis
MI: Mutual Information
SVR: Support Vector Regression
RF: Random Forest
KNN: K-nearest neighbor
VAE: Variational autoencoder
DAE: Deep autoencoder
NN: Neural network
ROC curve: Receiver operating characteristic curve
PR curve: Precision-Recall curve
AUC: Area under curve
