# Supplementary material for: Drug-induced cell viability prediction from LINCS-L1000 through WRFEN-XGBoost algorithm
Source: BMC Bioinformatics. 2021 Jan 6;22:13. doi: 10.1186/s12859-020-03949-w (PMC7788947; doi:10.1186/s12859-020-03949-w)
Supplement: Supplementary file 1 — Additional file 1. Supplementary Material contains supplementary figures and supplementary tables of the results in this study. [file 12859_2020_3949_MOESM1_ESM.pdf]

Drug-induced cell viability prediction from LINCS-L1000 through WRFEN-XGBoost algorithm

Supplementary Material

Supplementary Table1

The first 15 characteristic genes screened by the WRFEN algorithm

Subset Name	Number of genes selected	Top 15 key genes selected
CTRP-L1000-3h dataset	160	CDC25A;TSC22D3;ATP1B1;SQSTM1;CTSL;RUVBL1;CPSF4;CDKN1A;CENPE;BLCAP; ICAM1; RRP8; KDM3A; YTHDF1; RRS1
CTRP-L1000-6h dataset	193	MYC;POLE2;KDM3A;RGS2;BNIP3L;STX1A;TSC22D3;JUN;RRS1;MAT2A;CNPY3;TMEM109;MLEC;JMJD6;NUP62
CTRP-L1000-24h dataset	133	MRPL12;RGS2;NPC1;POLE2;TOP2A;POP4;PCNA;AURKA;CDH3;RPN1;CDK1;UBE2C;ALDOC;HSPB1;HDAC6
Achilles-L1000-96h dataset	252	GADD45A;UBE2C;CCNA2;MELK;TSPAN6;SMNDC1;PCNA;RBM34;WDR61;COL4A1;PGM1;ELOVL6;SOX4;TSC22D3;CTSD
Achilles-L1000-120h dataset	322	BIRC5;NFKBIA;DUSP4;CDK1;POLE2;SPDEF;TRIB1;INSIG1;FOXO4;CTSL;SMARCD2;FOXO3;BNIP3;PLS1;IGFBP3
Achilles-L1000-144h dataset	350	TES;IER3;CTSL;GNAS;PSMG1;ABCF3;SPAG4;B4GAT1;GLRX;TIMM22;CENPE;ABCC5;JMJD6;HOXA10;NCK1

Supplementary Table 2

XGBoost default parameters and best parameter combinations (Achilles-L1000 Series Model)

Parameter Name	L1000-Achilles-96h	L1000-Achilles-120h	L1000-Achilles-144h
learning rate	0.0100	0.0100	0.0225
gamma	0.2540	0	1.6190
max depth	6	6	7
min child weight	3	5	8
subsample	0.7014	0.5914	0.6071
colsample_bytree	0.9524	0.9048	0.3333
lambda	0.7365	0.5786	1.7157
Iteration times	4809	2984	3857

Supplementary Table 3

Comparison of the algorithm in this paper with other algorithms（Pearson correlation）

The screen used	This study	PCA-Lasso	PCA-SVR	FTest-RF	MI-KNN	VAE	DAE-NN
CTRP-L1000-3h(S2)	0.8392	0.7291	0.7166	0.7392	0.7791	0.7775	0.6789
CTRP-L1000-6h(S2)	0.6770	0.5985	0.5962	0.6343	0.6105	0.6812	0.6273
CTRP-L1000-24h(S2)	0.8859	0.8208	0.8176	0.8534	0.8706	0.8788	0.8660
CTRP-L1000-3h(S1)	0.7705	0.7210	0.7242	0.6488	0.7189	0.6981	0.6298
CTRP-L1000-6h(S1)	0.6239	0.5507	0.5545	0.5588	0.4991	0.5820	0.5105
CTRP-L1000-24h(S1)	0.8321	0.7887	0.7900	0.7942	0.8054	0.8289	0.8033
Achilles-L1000-96h	0.5893	0.5392	0.5266	0.5215	0.4334	0.5864	0.5045
Achilles-L1000-120h	0.5275	0.5036	0.5000	0.4800	0.3328	0.4643	0.4592
Achilles-L1000-144h	0.5348	0.5176	0.4975	0.4699	0.4066	0.5074	0.4822

Supplementary Table 4

Comparison of the algorithm in this paper with other algorithms（R^2）

The screen used	This study	PCA-Lasso	PCA-SVR	FTest-RF	MI-KNN	VAE	DAE-NN
CTRP-L1000-3h(S2)	0.6884	0.5179	0.5040	0.5362	0.5781	0.6043	0.4058
CTRP-L1000-6h(S2)	0.4578	0.3582	0.3423	0.3969	0.3148	0.4497	0.3692
CTRP-L1000-24h(S2)	0.7847	0.6736	0.6675	0.7262	0.7543	0.7660	0.7453
CTRP-L1000-3h(S1)	0.5803	0.5137	0.5153	0.4191	0.4828	0.4845	0.3557
CTRP-L1000-6h(S1)	0.3884	0.3012	0.2927	0.3098	0.1566	0.3137	0.1522
CTRP-L1000-24h(S1)	0.6922	0.6211	0.6211	0.6304	0.6414	0.6823	0.6174
Achilles-L1000-96h	0.3468	0.2905	0.2682	0.2684	0.1068	0.3224	0.2499
Achilles-L1000-120h	0.2782	0.2496	0.2379	0.2236	0.0240	0.1869	0.1736
Achilles-L1000-144h	0.2848	0.2657	0.2350	0.2176	0.1133	0.2460	0.2169

Supplementary Table 5

Comparison of the algorithm in this paper with other algorithms（Mean squared error）

The screen used	This study	PCA-Lasso	PCA-SVR	FTest-RF	MI-KNN	VAE	DAE-NN
CTRP-L1000-3h(S2)	0.021	0.033	0.034	0.032	0.029	0.027	0.041
CTRP-L1000-6h(S2)	0.054	0.064	0.066	0.060	0.069	0.055	0.063
CTRP-L1000-24h(S2)	0.018	0.027	0.027	0.023	0.020	0.019	0.021
CTRP-L1000-3h(S1)	0.029	0.033	0.033	0.040	0.035	0.035	0.044
CTRP-L1000-6h(S1)	0.064	0.073	0.074	0.072	0.088	0.071	0.088
CTRP-L1000-24h(S1)	0.025	0.031	0.031	0.030	0.030	0.026	0.032
Achilles-L1000-96h	1.477	1.604	1.655	1.654	2.019	1.532	1.696
Achilles-L1000-120h	1.391	1.446	1.468	1.496	1.880	1.567	1.592
Achilles-L1000-144h	1.307	1.342	1.398	1.429	1.620	1.378	1.431

Input: Training Sample $\boldsymbol{PertDT - S_{N*M}}$, Number of decision trees *TreeNum.*

Algorithm execution process:

1. In the training sample ***PertDT-S***, for each differential expressed gene ***g*** ∈***OriDEGs***:

   1.1 For each decision tree *t*:

   1.1.1 Bootstrap the training sample and then form the subset $\textbf{Sample}^*$.

   1.1.2 Construct a decision tree $\text{DecisionTree}_\text{t}$ by using $\textbf{Sample}^*$.

   1.1.3 Use the validation data to predict the results on $\text{DecisionTree}_\text{t}$ and the Pearson correlation coefficient is used to evaluate the prediction performance, which are recorded as the original error $error_t$.

   1.1.4 Add random noise to each gene ***g***, and use Pearson correlation coefficient to evaluate the prediction performance, which are recorded as $ERROR_{tg}$.

   1.1.5 Compute the importance value on $\text{DecisionTree}_\text{t}$ under gene ***g***.

$$\text{difference}_{tg} = ERROR_{tg} - error_t \circ \quad \text{(Formula S1)}$$

   End For.

   1.2 Obtain the average of all decision trees results under gene ***g***:

$$d_g^{\wedge} = \frac{1}{TreeNum} * \sum_{t=1}^{TreeNum} \text{difference}_{tg} \quad \text{(Formula S2)}$$

   1.3 Obtain the variance of all decision trees results under gene ***g***:

$$Var_g^2 = \frac{1}{TreeNum - 1} * \sum_{t=1}^{TreeNum} (\text{difference}_{tg} - d_g^{\wedge})^2 \quad \text{(Formula S3)}$$

   1.4 Calculate the importance of gene ***g***:

$$importance_g = \frac{d_g^{\wedge}}{Var_g^2} \quad \text{(Formula S4)}$$

   End for.

2. In the training sample ***PertDT-S***, for each parameter combination of α(the coefficient penalty) and λ(the ratio of the lasso penalty):

   2.1 Train the ElasticNet model under the specified parameter settings in the training sample ***PertDT-S***,.

   2.2 Use the validation data to predict the results on the ElasticNet and calculate the Pearson correlation coefficient to evaluate the prediction performance.

   2.3 Select the model with the highest Pearson correlation coefficient, and get the feature genes importance according to their weight coefficient.

   End for.

3. Calculate the feature importance according to Formula 2.

Output: The final feature importance.

Supplementary Figure 1

The flowchart of feature selection

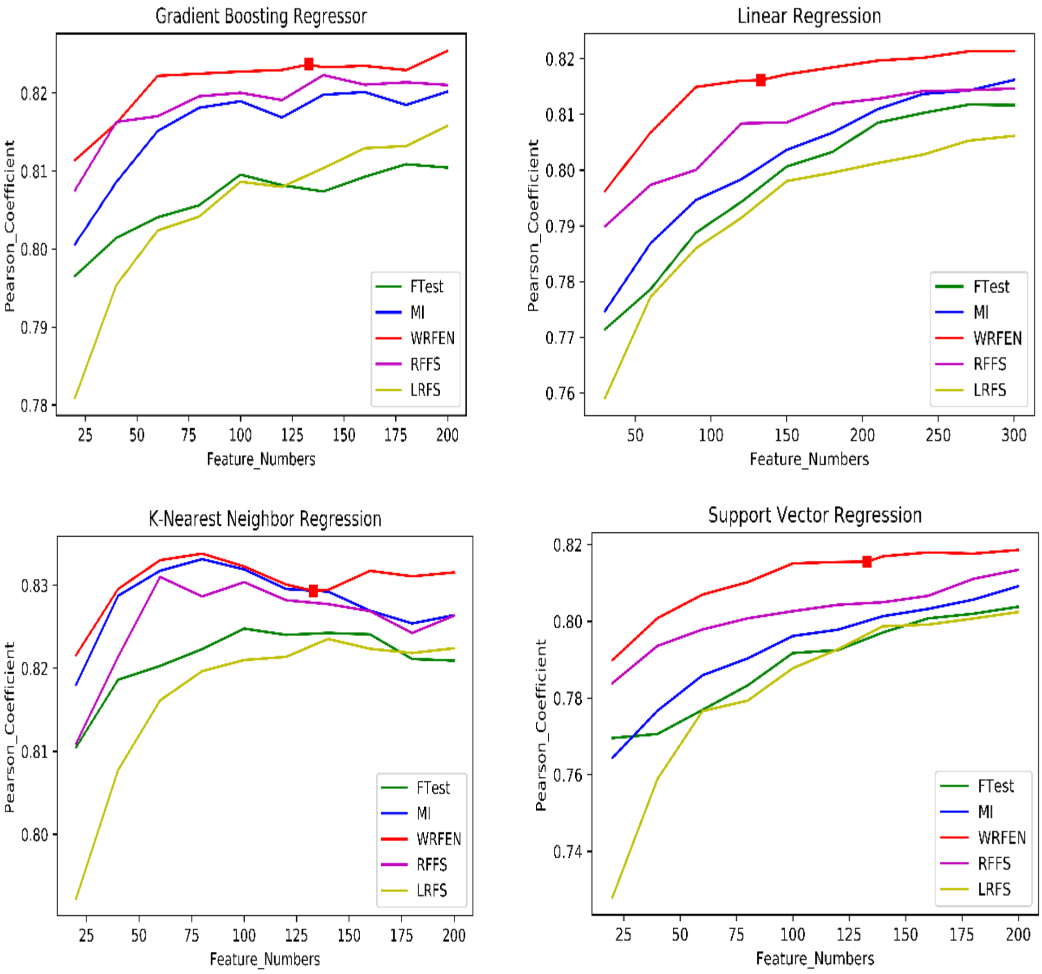

Supplementary Figure 2

Comparison of different key gene selection methods. “Ftest” refers to the joint hypotheses test. “MI” refers to mutual information. “WRFEN” refers to the method this study used. “RFFS” means using the Random Forest algorithm to select the key genes. “LRFS” means using the Linear Regression Model to select the key genes.

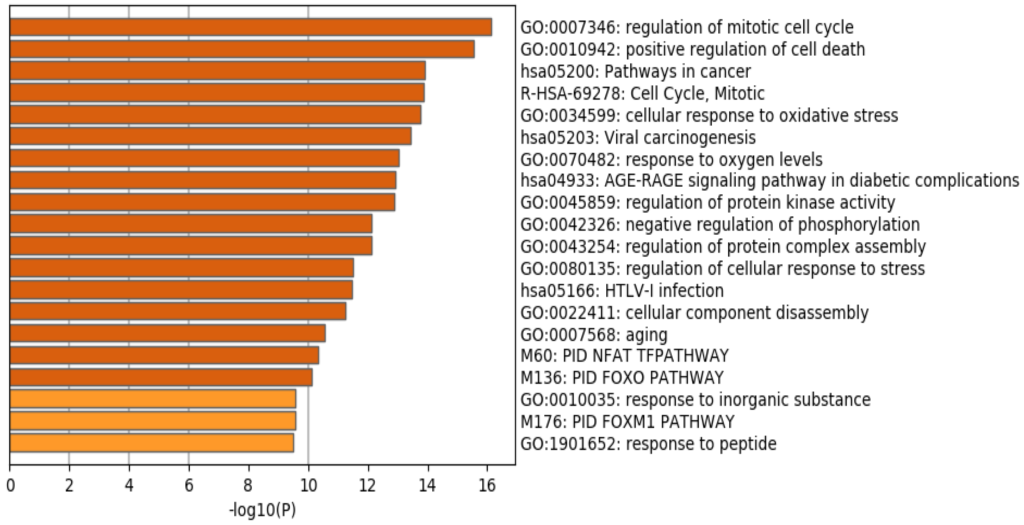

Supplementary Figure 3

Enrichment analysis of differentially expressed genes in the Achilles-L1000-96h dataset

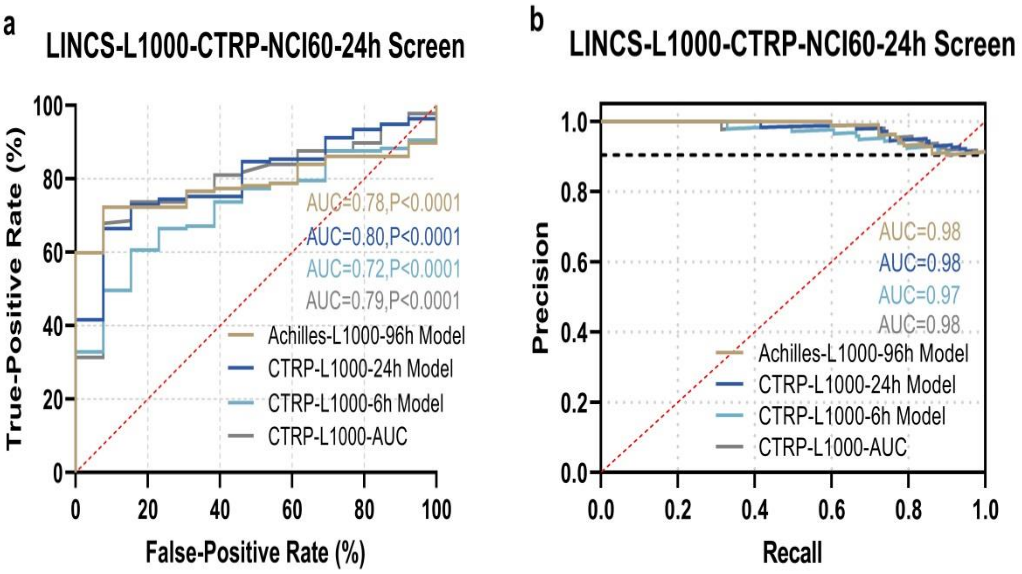

Supplementary Figure 4

ROC curve (a) and PR curve (b) of the model evaluation on LINCS-L1000-CTRP-NCI60-24h dataset

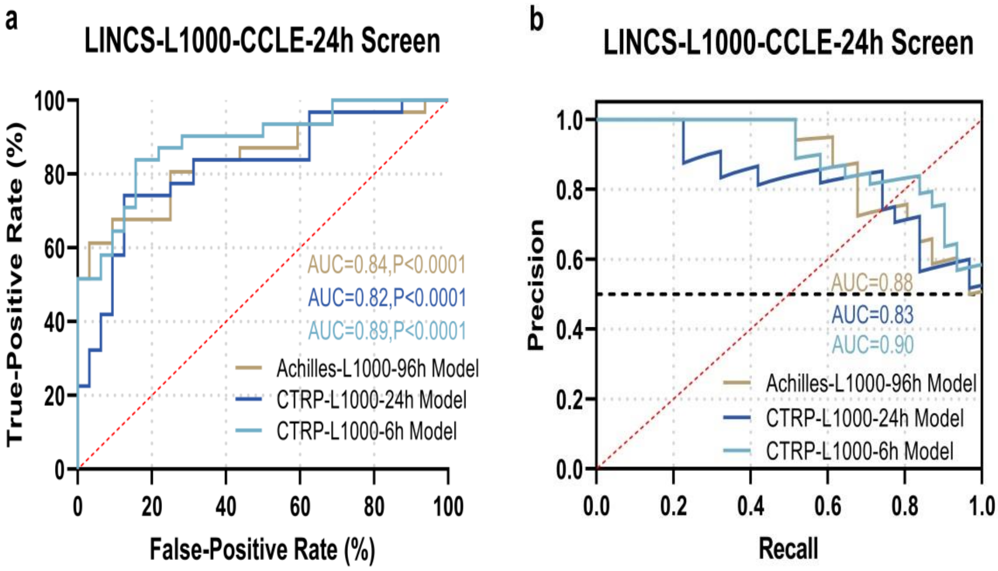

Supplementary Figure 5

ROC curve (a) and PR curve (b) of the model evaluation on LINCS-L1000-CCLE-24h dataset

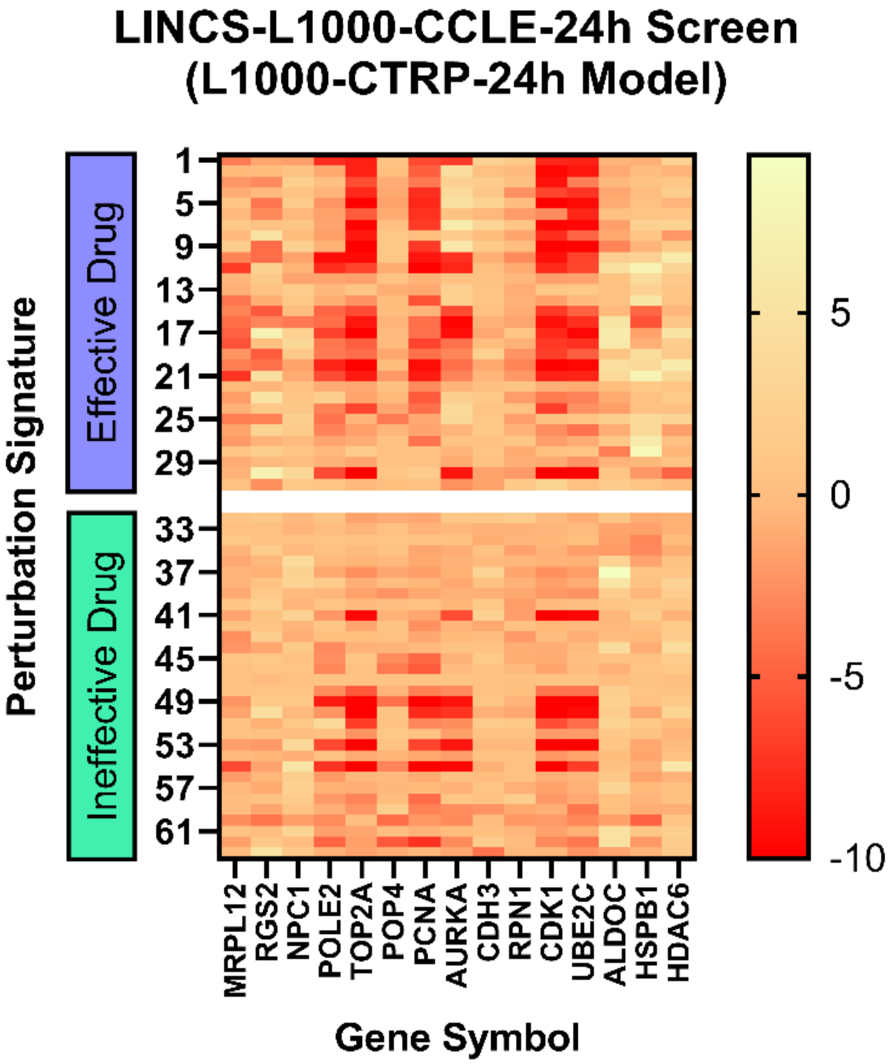

Supplementary Figure 6

Heat map of the first 15 genes of the LINCS-L1000-CCLE-24h dataset
